# Control of Frequency Combs with Passive Resonators

**DOI:** 10.3390/s23031066

**Published:** 2023-01-17

**Authors:** James Hendrie, Ning Hsu, Jean-Claude Diels

**Affiliations:** 1 School of Optical Science and Engineering, University of New Mexico, Albuquerque, NM 87106, USA; 2 Center for High Technology Materials, University of New Mexico, Albuquerque, NM 87106, USA

**Keywords:** intracavity phase interferometry, laser sensors, precision sensing, inertial sensors, gyroscopes, ultrafast

## Abstract

Tailored optical frequency combs are generated by nesting passive etalons within mode-locked oscillators. In this work, the oscillator generates a comb of 6.8 GHz with 106 MHz side-bands. This tailored comb results from the self-synchronized locking of two cavities with precision optical frequency tuning. In this manuscript, it is demonstrated that these combs can be precisely predicted utilizing a temporal ABCD matrix method and precise comb frequency tuning by scanning over the D1 transition line of ${}^{87}$Rb and observing the fluorescence.

## 1. Introduction

Sensors have traditionally been based on narrow band continuous lasers. Time standards are now based on frequency combs ever since the Nobel prize was awarded to Jan Hall and Ted Hänsch for frequency combs. This has brought the knowledge that, contrary to popular belief, trains of ultrashort pulses can be used for precision measurements. There are numerous types of sensors based on frequency combs requiring accurate determination of the position and spacing of their teeth. Standard mode-locked cavities generate frequency combs whose dynamics and stabilization have provoked discourse and research throughout the ultrafast community since their conception [1,2]. There has been extensive work done in the area of understanding and categorizing these combs and the aspects of the cavities that create them [3]. Stabilization of combs using extra-cavity Fabry–Perot’s has also been thoroughly investigated [4,5]. In this work, a *passive* cavity is nested within an *active* parent cavity resulting in a comb of two frequencies whose dependencies result in a wholly unique set of intra-cavity dynamics.

Previously, researchers have set out to broaden the spectrum of frequency combs [6] and flatten their amplitude profile [7] for various applications, mostly having to do with spectroscopy [8,9] and stabilization [5]. Others have utilized these combs for the creation of stable clocks [10,11], and even to attempt to achieve reliable quantum memory with quantum bits [12]. The technique described in this paper is particularly useful in the context of ultrasensitive sensors based on intracavity phase interferometry [13], and sensors based on atomic transitions.

All of the aforementioned projects relied upon combs from the typical Saturable Absorber (SA) Titanium Sapphire cavities whose frequencies are set by the repetition rate of a single oscillating pulse. These cavities being typically of the order of meter length, the mode spacing is of the order of 100 MHz. It will be shown here how tooth comb spacing in the GHz range can be achieved in these long cavities.

By purposefully modulating the frequency comb itself, it is possible to move beyond the idea of a comb as simply a fixed ruler, but rather as a dynamic tool that can be tuned to various atomic resonances or to focus photon energy to just a few desired frequency modes. This is achieved by inserting in the cavity a passive optical resonator such as a Fabry–Perot etalon.

Adding an etalon in an active cavity is a standard technique for mode selection, or for tuning the wavelength of a single mode laser. A theoretical study of the influence of a Fabry–Perot inserted in the cavity of a mode-locked fiber laser [14] concludes only on a tenfold broadening of the individual pulses. One motivation for inserting a Fabry–Perot in a mode-locked laser cavity has been mainly to achieve high repetition rates by harmonic mode-locking. Yoshida et al. [15] achieved a repetition rate of 40 GHz with an etalon inserted in a unidirectional mode-locked ring laser cavity. A passive etalon in—or coupled to—an active cavity has been used to achieve a certain amount of mode cleansing in harmonically mode-locked fiber lasers [16,17]. Sophisticated electronic stabilization was required to maintain an integer ratio between modes of the Fabry–Perot and of the laser cavity. Very high finesse Fabry–Perot—up to 10,000 [18]—were used in these applications. A different approach consists in inserting in a mode-locked laser a very low finesse etalon (uncoated or antireflection coated glass window). It was shown in [19] that each pulse of the original pulse train was replaced by a bunch of pulses with a period related to the etalon round-trip time. The latter period was observed to be a submultiple of the laser cavity round-trip time, a property attributed to gain dynamics [19]. It will be shown here in Section 2, and modeled in Section 3, that the pulse periods dependence on laser cavity length and etalon are explained by the coupling between the long and short cavities. The low Q short etalon acquires the finesse of the long laser resonator. This property has direct impact on the sensitivity of sensors based on intracavity phase interferometry [13], because the dispersion of the etalon affects each mode of the frequency comb [20]. Depending on its sign, the resonant dispersion affects the phase velocity of the intracavity pulses circulating in the laser, resulting in“fast” or “slow” light. The “fast” light enhances the phase sensitivity of a sensor [20,21,22,23].

Section 2 provides a clarification of definitions used in this paper and a brief description of the generalized cavity layout. A method for modeling the dynamics of these pulse bunches using modified ABCD time matrices is explained in Section 3. Section 4 then presents the conceptual and experimental evidence of comb locking and cavity synchronization. A series of experiments using the D1 resonance line of ${}^{87}$Rb to demonstrate comb dynamics and the precision by which these dynamics can be predicted and tuned is given in Section 5. Finally, a discussion of possible future applications and a summary of conclusions found throughout this work are given in Section 6.

## 2. Description of the Nested Cavities and Their Frequency Combs

**Figure 1:**
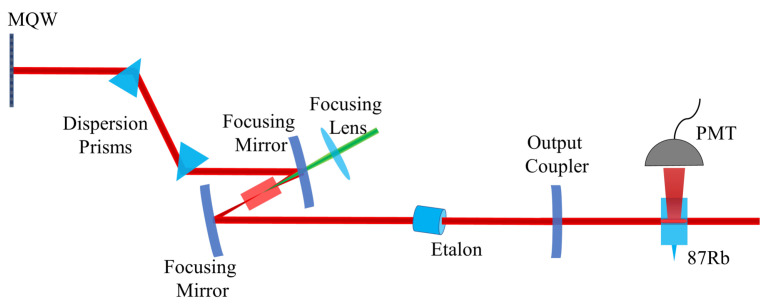
A schematic of a Ti:Sapph oscillator, pumped by a 532 nm source. A simple etalon nested in the oscillator is depicted at an exaggerated angle (typical angles are from 0 mrad to 20 mrad). One end mirror uses a standard semiconductor multiple quantum well (MQW) to assist mode-locking. The output of this cavity is sent to a ${}^{87}$Rb vapor cell for the characterization experiments that are described in a later section. A Photo Multiplier Tube (PMT) is used to collect the fluorescence of the vapor cell as the laser elements are scanned.

**Figure 2:**
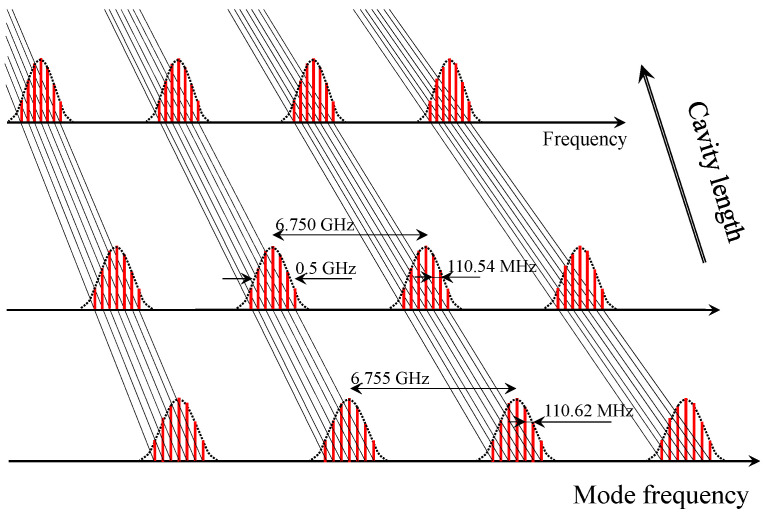
Laser cavity length dependence of the comb produced by the nested design depicted in Figure 1. For a particular cavity length (of the order of 2.71 m), laser cavity modes with a spacing of 110.62 MHz are identified (bottom of the figure). This structure repeats periodically every 6.755 GHz, a frequency that is sightly smaller than the etalon mode spacing of 6.821 GHz, since the etalon is 15.12 cm long with an index of 1.4534 at 20C. As the laser cavity length is increased, all mode spacings shrink in the same proportion.

The cavity configuration is shown in Figure 1. The main (active) cavity is a standard Ti:Sapphire laser mode-locked via a multiple quantum well (MQW) saturable absorber. A 15.12 mm thick fused silica (uncoated) etalon is inserted in the 2.71 m long laser, acting as a passive “nested” cavity. Two fused silica Brewster angle prisms spaced by 1 m are used to compensate for the dispersion of the 4 mm long Ti:sapphire gain crystal. The gain crystal is pumped by a 5W Verdi (Coherent) frequency-doubled vanadate laser. A 3% transmission output coupler is mounted o a motorized translation stage to tune the laser cavity length. It may seem as an anathema to insert a frequency filter in a mode-locked laser. The first action of the Fabry–Perot is to tune the optical frequency of the modes, just as in a cw laser. Only radiation at transmission resonances of the Fabry–Perot can survive. In the cw case, the radiation builds up in the etalon at resonance, in order for the transmission to be unity. With pulses shorter than the etalon optical thickness, at each laser cavity round-trip a pulse will shed energy by reflection at the interfaces, to a secondary pulse delayed by the etalon round-trip time. That satellite pulse is re-injected in the main cavity, gaining energy from the parent pulse at each round-trip, and shedding energy to the next satellite. This process leads to the generation of a pulse bunch of repetition rate dominated by the etalon thickness, which repeats at the round-trip time close to that of the main laser cavity. In a frequency domain description, a comb with small teeth spacing is nested in a structure with wider spacing. The exact spacing between these sets of teeth is a function of the laser saturated gain, and the reflectivity of the Fabry–Perot surface. One might think that the larger and smaller teeth spacing are unrelated. Instead, they are in a constant ratio, as has been experimentally demonstrated by scanning the length of the laser cavity [19,20]. The overall picture that emerges is sketched in Figure 2.

**Figure 3:**
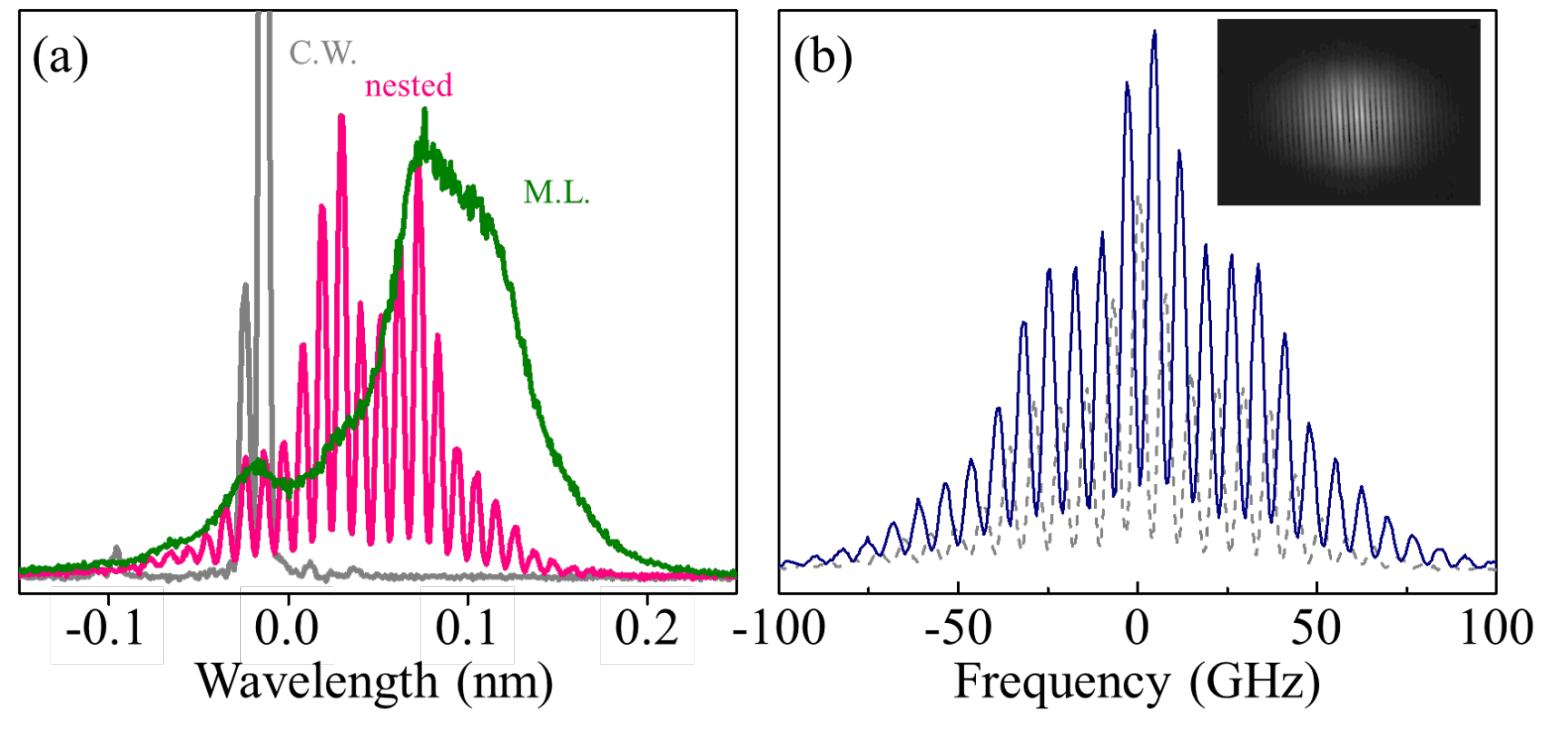
Single grating (1200 grooves/mm) spectrometer is used to resolve the 6.8 GHz free spectral range of the etalon. (**a**) shows how the spectrum of the laser is modified in different configurations. The green line is a mode-locked spectrum in the typical Ti:Sapph cavity without an etalon. The gray line is the continuous wave spectrum with a nested etalon, and the pink line is the mode-locked spectrum with a nested etalon. In (**b**) the solid blue line and the dashed line show how the optical spectrum visibly shifts as the etalon internal angle is tuned by about 10mrad. The inset of this plot is the raw data from the CCD showing the clear contrast of the Fabry–Perot etalon’s modulation of the optical spectrum.

A qualitative demonstration both of the effect of a nested etalon in the frequency domain as well as its frequency tuning capabilities is performed with a simple single grating and a CCD. This configuration provides a *direct* visualization of the broader GHz structure, visualization that is not possible with traditional RF spectrum analyzers that look only at the *difference* between optical modes. The spectral imaging setup comprises a grating (1200 grooves/mm) and a focusing lens that images the spectrum onto a CCD. The first-order diffraction angle is about 0${}^{\circ }$ when the incident angle is at 85${}^{\circ }$. Figure 3a shows an example of a nested comb structure (red) resolved using this technique compared both to a cw spectrum with an etalon present (grey) and a standard mode-locked spectrum without a nested etalon (green).

The instrumental resolution in this configuration is about 5.5 GHz, which allows the Fabry–Perot etalon modes to be partially resolved. A central frequency shift is observed when the laser cavity is with (red) or without (green) an etalon. The dashed gray spectrum results from the mode selection function of etalon inside the cavity, as the modes in continuous-wave lasing are lined up with the modes in mode-locked lasing.

Figure 3b displays the nested comb spectra when the etalon is tuned to different angles. A shift of about half of the tooth spacing, ≈3.5 GHz, can be observed between the initial (blue) and the tuned (dashed) spectral traces. This provides a direct and convenient approach to monitor spectral dynamics.

The modes related to the etalon as well the modes related to the parent are equally spaced. However, the extended frequency groups do not merge into each others. That is, the repetition rate of a pulse bunch in the parent cavity is not a perfect sub-multiple of that of a pulse in the nested etalon. This fact is pointed out experimentally in Section 4.

A tooth of the tailored optical comb can be mathematically described by:

(1) $$f_{M,N}=f_{\mathrm{Offset}}+M\times f_{FP}+N\times f_{\mathrm{Parent}},$$

where $f_{\mathrm{Offset}}$ is the frequency by which the comb is shifted from overlapping with the 0 frequency, $f_{FP}$ is the repetition rate of the nested etalon, $f_{\mathrm{Parent}}$ is the bunch repetition rate of the parent oscillator, *M* is the mode index of the etalon, which will be discussed further in Section 5, and must be a real, positive integer, and *N* is the mode index of the parent cavity that is an integer from a negative value to a positive value. The range of *N* corresponds to the number of frequency teeth in each frequency group.

## 3. Analysis of Pulse Bunch Formation and Propagation

In this section, the dynamics and the evolving nature of the tailored frequency comb are studied using time transfer matrix method. The problem is first simplified by considering only the parent cavity (Ti:Sapphire) and reproducing pulse characteristics that were experimentally observed. This validation is followed by a complete nested cavity simulation and by comparing different aspects of the pulse properties to physical measurements. In the final part of this section, the implication and limitation from this study is discussed.

The peculiar properties of a tailored optical frequency comb were first reported by Masuda et al. [19]. In their model, the process of balancing saturable gain and loss in an active cavity leads to the formation of a symmetric, stable pulse bunch as confirmed by the experiments. Their approach does not include the influences of pulse propagation and therefore cannot explain the 10 ps pulse width they experimentally observe. This study includes the propagation effects and their contributions to the formation of the tailored frequency comb. By using a time transfer matrix method, all the pulse properties including energy, pulse width, and chirp are concurrently taken into account.

In a time cavity, the dispersion from the prisms pair can be described as [24]

(2) $$\left[\begin{array}{cc} A & B\\ C & D \end{array}\right]=\left[\begin{array}{cc} 1 & \frac{L_{g}}{c}[2\frac{dn}{d\Omega }{|}_{\omega_{}}+\omega_{}\frac{d^{2}n}{d\Omega^{2}}{|}_{\omega_{}}]-\frac{\omega_{}}{c}\left(4L+\frac{L_{g}}{n^{3}}\right){\left(\frac{dn}{d\Omega }{|}_{\omega_{}}\right)}^{2}\\ 0 & 1 \end{array}\right],$$

where $L_{g}$ is the length of propagation inside the prisms, *L* is the separation between two prisms *n* is refractive index, and ω refers to the central angular frequency. In the first line, second column of the transfer matrix, the first term in brackets is simply the GVD dispersion of glass, while the second term is the angular dispersion. The self-phase modulation matrix is

(3) $$\left[\begin{array}{cc} 1 & 0\\ \frac{1}{f} & 1 \end{array}\right]=\left[\begin{array}{cc} 1 & 0\\ \frac{\lambda \tau_{g0}^{2}}{8\pi n_{2}I_{0}\ell_{Kerr}} & 1 \end{array}\right],$$

where $n_{2}$ is the nonlinear refractive index, $I_{0}$ is the intensity of the pulse, $\ell_{Kerr}$ is the length of propagation in the Kerr medium and $\tau_{go}$ the (Gaussian) pulse duration. Unlike the space transfer matrix, the time transfer matrix is intensity dependent, and needs to be recalculated at each round trip. In the simulation, the $n_{2}$ of sapphire is $8\times 10^{-16}$ cm${}^{2}$/W, the refractive index *n* at 780 nm is 1.76 and the crystal length is 0.6 cm. The compensating dispersion is provided by a pair of fused silica prisms with 1 m in tip-to-tip separation. We keep the 106 MHz overall repetition rate the same between the single pulse and pulse bunch cases. For an individual pulse within a pulse bunch, we use a pulse energy of about 10 nJ, a pulse width of 10 ps, and a peak power of 100 W for a 0.1 mm beam size. The intensity, therefore, is about 10 kW/mm${}^{2}$ = 1 MW/cm${}^{2}$. For the single pulse case, we use a pulse energy of about 10 nJ, a pulse width of 100 fs, and a peak power of 0.1 MW for a 0.1 mm beam size. Here the intensity is about 10 MW/mm${}^{2}$ = 1 GW/cm${}^{2}$. These are initial parameters as they are recalculated per roundtrip. A saturable gain is included to balance out the loss.

**Figure 4:**
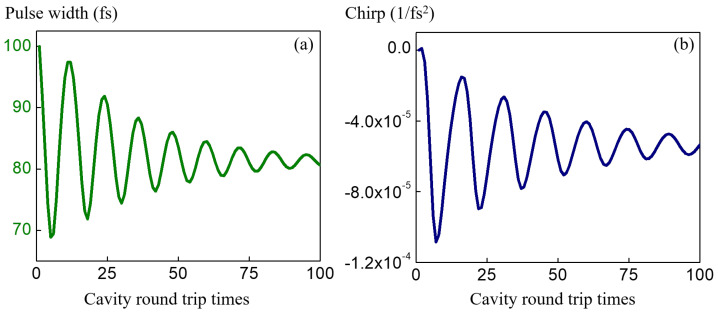
ABCD time transfer matrix method is applied to simulate single pulse (Ti:Sapphire cavity alone) circulating in the cavity. The convergence of the (**a**) pulse width and the (**b**) chirp coefficient are showing the compensation of the self phase modulation and dispersion in a stable time cavity.

The simulation result for a simple Ti:Sapphire cavity is presented in Figure 4. In this case the balance between the self-phase modulation and the dispersion can be seen in panels (a) and (b), where the pulse width and chirp stabilize to a constant. The pulse width is about 100fs, which is typical for the mode-locked Ti:Sapphire laser mode-locked by a standard semiconductor multiple quantum well mirror. In the nested cavity scenario the etalon acts as a loss source to the individual pulses, which changes the dynamics of the energy and gain.

**Figure 5:**
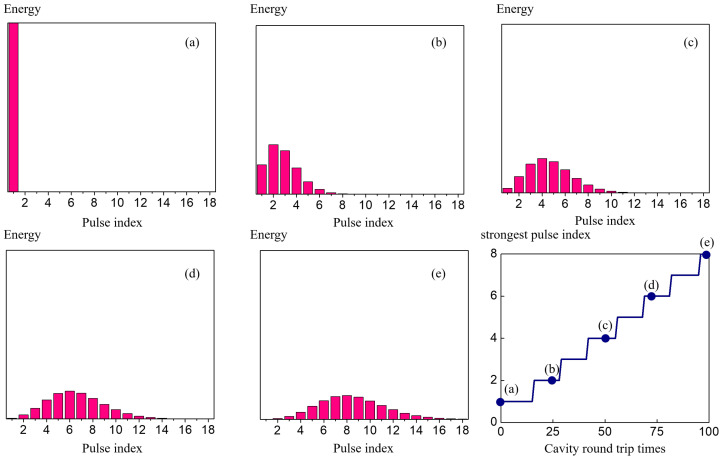
A single pulse starts to develop into a pulse bunch when an etalon is placed in the parent cavity. Panels (**a**–**e**) show the different frames of pulse bunch developing when a single pulse is propagating at (**a**) 0, (**b**) 25, (**c**) 50, (**d**) 75, and (**e**) 100 round trips in the nested cavities. A symmetric pulse bunch is formed where about 15 pulses sits under the bunch envelope. The lower right panel records the pulse position within the bunch with the highest energy along the propagation. The slope defines the envelop velocity.

**Figure 6:**
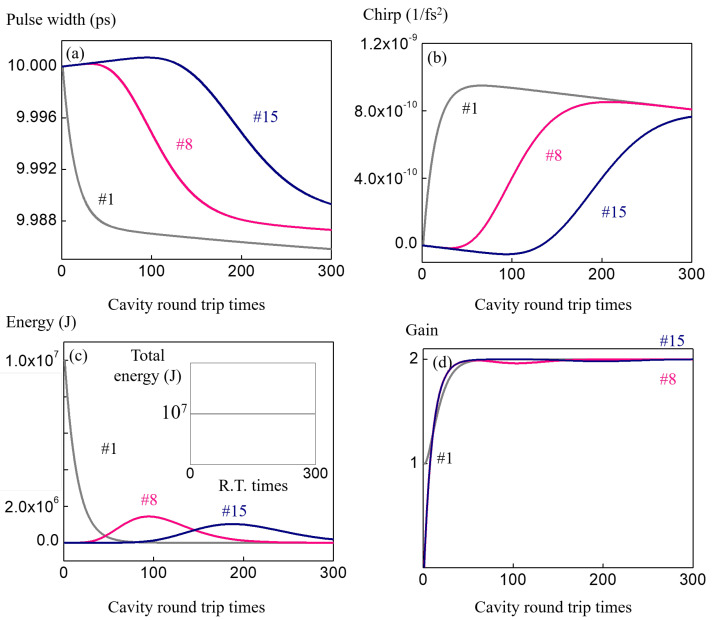
This figure shows the dynamics of the individual pulses (15 in total) in the pulse bunch in Figure 5. For clarity, only pulses index number 1 (first pulse, in gray), 8 (central pulse, in red), and 15 (last pulse, in blue) are plotted, where distinct behaviors can be observed from (**a**) pulse widths, (**b**) chirps, (**c**) energies, and (**d**) gain. In (**a**,**b**), the energy differences cause the balance (gray) or imbalances (red and blue) between the self phase modulation and the dispersion, which results in pulse widths change and differences in the sign of the chirp coefficients. Panel (**c**) shows the energy exchange from the three pulses. Due to the direction of coupling, the energy of the first pulse drops and the central and the last pulses grow. A more complete picture of energy trading can be seen from Figure 5. When the pulse bunch is well developed into a symmetric, constant shape, the gain and loss are balanced for every pulses, as shown in the panel (**d**).

The simulation results of the nested cavity with an etalon(reflectivity of 0.07) are shown in Figure 5 and Figure 6. The former shows the formation of a bunch with constant shape traveling at the bunch speed, and the latter decomposes the bunch into individual pulses to show the pulses width, chirp coefficient, energy, and gain.

In Figure 5a–e, for easier visualization, column plots are used to show the energy in column height and shapes and widths of the pulses are discussed in Figure 6. The horizontal axis is the index of the pulses. Panel (a) shows the initial condition where only a single pulse exists in the cavity. During the 100 roundtrip propagations, only the first 18 pulses show significant change in their energies. As the number of roundtrips increase, the concentration of the energy is pushed forward to the pulses with higher indices. The evolution of the pulse bunch can be seen from panels (a)–(e), and once the envelope is formed, it travels at the bunch envelope velocity. The lower right panel plots out the index of the pulse where it has the maximum energy during propagation, which corresponds to the peak of the bunch. The bunch envelope speed can be calculated by finding the slope of the blue solid line.

The dynamics of the individual pulses under the bunch enveloped is presented in Figure 6. Four panels are plotted in the same format as in the single pulse scenario in Figure 4 for comparison. During the first 100 roundtrips, only 18 of pulses are involved. For the sake of clarity, only the first (number 1), the middle (number 8), and the last (number 15) pulses inside the bunch envelope are presented, where distinct characteristics are observed. During the first 100 roundtrips, the first few pulses contain most of the energy, which help to balance the negative dispersion by stronger self-phase-modulation. On the other hand, the pulses with higher index temporarily see an unbalanced time transfer matrix, which induces broadening in width and accumulation of chirp. As the pulse bunch travels, the first few pulses lose their energy and see the unbalanced matrix, and for the higher index pulses such as number 8 and 15, their higher intensity start to help balance the negative dispersion. An apparent reversal of the slopes for number 8 and 15 can be seen at roundtrips higher than 100. In Figure 6c, the transfer of energy between pulses can be seen, which also supports the interpretation of balanced and unbalanced self-phase modulation matrices. The inset shows the energy sum of all the pulses, which is constant because of the loss free nature. The constant shape of the pulse bunch can be explained by the stabilization of the gain for all the pulses as shown in the Figure 6d. The overall propagation can be described as quasi-stable, where the pulse envelope is developing into a constant shape, while the individual pulses evolve and cycle from unbalanced, balanced, and to unbalanced again in time.

This time transfer matrix method provides the insight about the influence of adjacent pulses coupling and time propagation on the pulse bunch formation. After introducing a nested etalon, a pulse bunch envelope forms and maintains a constant shape even though the individual pulses become unstable. The scope of this simulation is limited to Gaussian-shaped pulses because it is based on time transfer matrix. Future study would involve the space-time propagation without Gaussian beam and pulse approximation.

## 4. Auto-Synchronization of Parent and Nested Cavity

**Figure 7:**
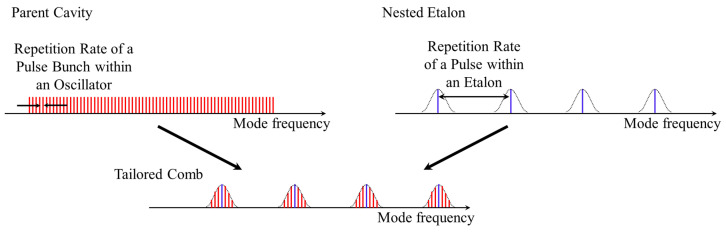
This figure shows the standard mode-locked comb, on the left, and the resonant FP spectrum, on the right coming together to form the tailored comb, on the bottom.

**Figure 8:**
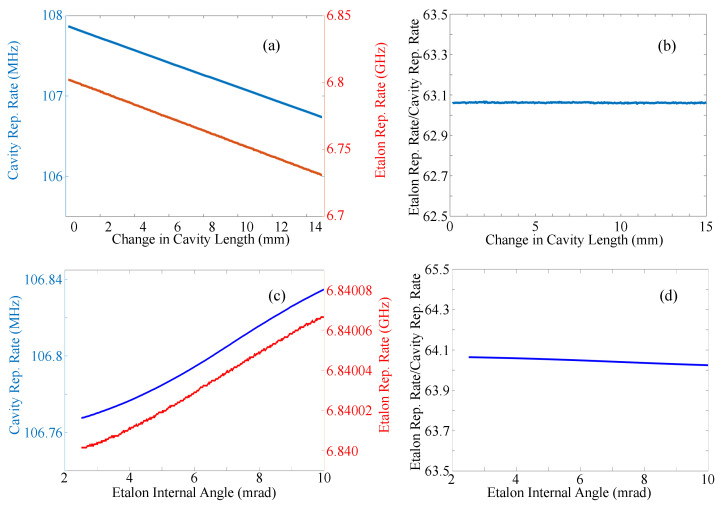
(**a**) Plot of the repetition rate of a single pulse within the nested etalon (blue line) and that of the pulse bunch in the larger cavity, as function of the laser cavity length. (**b**) Ratio of the two frequencies. (**c**) Plot of the two repetition rates of the parent cavity and the etalon taken simultaneously while the angle of the nested etalon is scanned. (**d**) Ratio of these two frequencies.

While the previous section focused on a description of the cavity dynamics in the time domain, this section will focus on the interplay between the combs generated by these bunches in the frequency domain. Looking at a tailored comb as two separate combs nested together, one can say that the two combs are uniquely locked and therefore synchronized. As shown in Figure 7, there are two main frequencies that make up these two combs, the repetition rate of a pulse within a passive etalon and the repetition rate of a pulse bunch within an active oscillator. The locking of these frequencies is evidenced in Figure 8 which records their value, part (a), and their ratio, part (b), as the length of the parent cavity is scanned.

Too often, the notion of group velocity is associated with the frequency derivative of the *k* vector in a dispersive medium. However, it has been demonstrated that this traditional definition of group velocity does not apply to media with saturable gain/absorption [25]. In a fiber laser, energy exchanges between the leading and trailing edge of the envelope determine its velocity [26]. The situation is similar in the laser with intracavity etalon: at each round-trip, a small portion of energy of each pulse of a bunch is transferred to the next one. The resulting reshaping of each bunch amounts to a reduction of its velocity. The velocity reduction results to an increase of the pulse round-trip time in the Fabry–Perot and laser cavity [19]. This explains the proportionality between repetition rated within the etalon or within the cavity as illustrated in Figure 8a. The ratio of pulse repetition rate within the bunch to bunch repetition rate as shown in Figure 8b shows a slope of 0, meaning that the frequencies are changing at an equal rate and are locked together. The ratio of the two frequencies is close to, but slightly offset from, an integer. As the cavity length is scanned, the laser properties are adapted such that the various repetition rates change identically and are never an integer multiple of each other.

If, instead of adjusting the parent cavity length, the angle of the nested passive cavity is scanned by sub micro-radian values, a similar result is found as shown in Figure 8c,d. As in (a) and (b), the frequencies are plotted in part (c) and the ratio in part (d). Again, the ratio is seen to be flat and offset from an integer.

**Figure 9:**
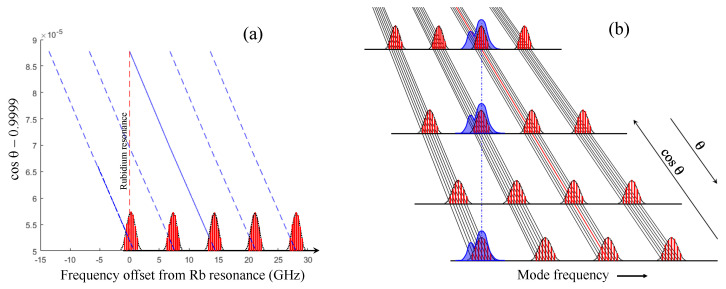
Evolution of the frequency of the mode comb teeth versus etalon angle. (**a**) The position of the center of each group of modes is plotted versus the cosine of the internal angle θ of the Fabry–Perot. θ=0 corresponds to normal incidence. The resonance the modes with the D line of ${}^{87}$Rb is taken as frequency origin. (**b**) Sketch illustrating the tuning of the comb structure with the Fabry–Perot angle. The purple area indicated the spectrum of the rubidium line.

The dependence of the mode frequency on the etalon angle is depicted in Figure 9. The fluorescence of the D line of ${}^{87}$Rb is used as frequency reference. As expected, for a Fabry–Perot, its resonance frequency varies linearly with the cosine of the internal angle cosθ. Different modes of the Fabry–Perot come into resonance as the angle of the Fabry–Perot is increased (Figure 9a). When the individual laser modes are recorded, the picture that emerges is that sketched in Figure 9b. All modes change proportionally, such that lines connecting corresponding modes converge.

The observed locking of these aspects of tailored combs can be called *self-stabilization* since the mode-locked oscillator in the nested case dynamically adjusts to maintain a fixed ratio of pulse bunch and individual pulse dynamics. and is thus *stabilizing* its frequency without the influence of an outside active stabilization method. Such passive stabilization could greatly decrease the need for complicated electronic stabilization systems in timing measurements and references.

## 5. Tunability of the Teeth of the Comb

This section will demonstrate the precise tuning capabilities and predictive nature of tailored combs. First, an experimental visualization of the Fabry–Perot etalon modes in the optical domain will be shown. Then this spectrum shall be scanned over a vapor cell of ${}^{87}$Rb to show the coincidence of these optical modes with a precisely known atomic transition. Finally, this coincidence of modes and transitions will be predicted mathematically and shown to be accurate.

### 5.1. Atomic Spectroscopy with the Tailored Frequency Comb

**Figure 10:**
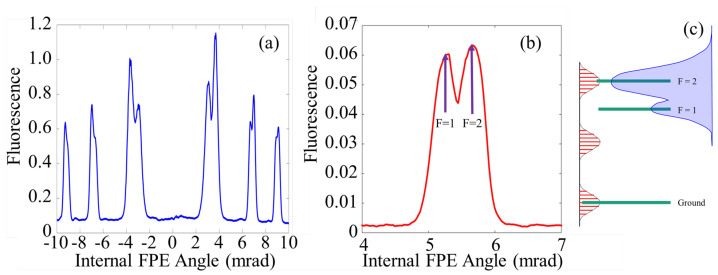
(**a**) Plot of the fluorescence intensity as the etalon’s angle is tuned 20 mrad. At 0 mrad, the etalon is in normal incidence to the larger cavity, and therefore, prevents mode-locking from occurring. It is not abnormal to see some fluorescence in this location, but that fluorescence is related to the instability of the laser in this region. This region of instability is typically ±2 mrad from 0 mrad. (**b**) Zooming into the first fluorescent order of (**a**). The two maxima are labeled as F = 2 and F = 1 with the associated measured values for the angles at which they occur. These values are picked as the rough center of the average envelope of the peaks and the labels are the predicted state each maxima is associated with. (**c**) A cartoon depiction of an rf comb aligned to the energy levels of the D1 line of ${}^{87}$Rb.

In this subsection, the resolved modes of Figure 3 are used to excite ${}^{87}$Rb fluorescence. By changing the angle of the Fabry–Perot etalon, the optical modes are scanned over the Rb vapor, and fluorescence peaks appear as shown in Figure 10a. Each time a fluorescent peak occurs, only a single Fabry–Perot mode is interacting with the atoms, though many frequency modes associated with the parent cavity are involved. Each time a resonance is achieved, the optical spectrum is reproduced. That is to say, for each peak in Figure 10a, the same optical spectrum is observed. Figure 10b shows the first order peak zoomed in. The wavelength value of the peaks identified as F = 1 and F = 2 are known via the notes of Dr. Daniel Steck [27] which contain the values of the frequency separation between the excited states of ${}^{87}$Rb as well as a tabulation of the coupling coefficients of the F = 1 and F = 2 lines.

These known values can be used to calibrate the abscissa of Figure 10 through

(4) $$\cos\left(\theta \right)=\frac{\lambda M}{2nd},$$

where *M* is the mode of the Fabry–Perot, λ the wavelength, *n* the index of refraction, which is taken to be n=1.453432 for λ = 795 nm at 23.2 ${}^{\circ }$C and an etalon thickness of *d* = 15.12 mm.

Figure 10c. shows the radio frequency (rf) (radio frequency (rf) combs are the combs that can be measured directly on the available rf spectrum analyzer. These combs can be described in two ways. The first is that an rf comb is the beating of the teeth of its optical counter part. A more physical description is that the rf comb is measured as the time between pulses read by the 30 GHz detector connected to the 13 GHz analyzer. That is to say the frequency peaks that make up the rf comb are a direct measure of the frequency at which the pulses reach the detector) comb next to a cartoon diagram of the D1 line of ${}^{87}$Rb. There are two reasons for using the rf domain. First, it is directly measurable on a spectrum analyzer allowing for the viewing of each individual laser mode rather than the overall modulation of the spectral profile, shown in Figure 3. Secondly, the rf comb is more helpful in describing how modes scan across these atomic transitions. In this rf domain, fluorescence is observed when one rf tooth aligns with the ground state and one aligns with an excited state. As the angle of the etalon is scanned, the spacing of these teeth changes by hundreds of megahertz until it aligns with the other excited state. Since the parent cavity discussed in this paper has a repetition rate on the order of 100MHz, multiple laser frequency modes can come into resonance as the internal angle is scanned over a single peak.

### 5.2. Calibration of the Frequency Comb

**Figure 11:**
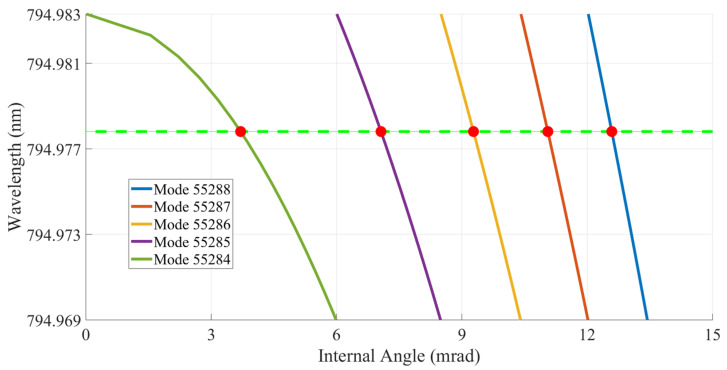
The red points are plotted to mark the angle at which each etalon mode coincides with the resonance line of ${}^{87}$Rb D1 line F = 2 state. This calculation is done for a room temperature corresponding to 23.4 °C. These points directly aligned to the F = 2 fluorescence peaks in Figure 10a. The plotted curves depict the frequency sweep of each etalon mode as the internal angle of the etalon is tuned. The green dashed line marks the value of the F = 2 resonance.

Using Equation (4), it is possible to calculate the mode and frequency of the Fabry–Perot etalon that is on resonance with the vapor cell in Figure 10a. The results of this calculation are shown in Figure 11 where the exact etalon modes are calculated and shown in the legend and the frequencies of those etalon modes are plotted. The green dashed line serves as a reference to the F = 2 state resonance of the D1 line of ${}^{87}$Rb. The first 3 predicted points in Figure 11 correspond exactly to the positions of the first three fluorescence locations in Figure 10a. This result proves the highly predictable nature of the tailored frequency comb’s tuning.

Since these resonance locations are predicted by the Fabry–Perot etalon modes, and not the MHz side-bands, there are GHz gaps between the MHz frequency clusters within the frequency domain picture. The advantage of this is that while these mode-locked sources are capable of large bandwidth spectrum, it is also possible to tailor a comb such that only specific transitions are probed with no excess fluorescence associated with nearby transitions. To put it another way, this is proof that a frequency comb can be specifically tailored such that the photon energy exists only in specific frequency regions.

## 6. Conclusions

Nested cavities consisting of 6.8 GHz combs with 106 MHz side-bands are demonstrated and characterized in a variety of experiments. A method for modeling pulse propagation inside these cavities and the resulting tailored combs have been defined and broken into their two major contributing parts. Furthermore, these cavities have been shown to demonstrate what has been defined as auto-synchronization through passive means via the locking of these parts.

The control of the comb structure is invaluable for ultrasensitive sensors based on intracavity phase interferometry [13]. It has been shown that the sensitivity of such sensors is proportional to the mode spacing of the comb, mode spacing that can be tens of GHz through the insertion of an etalon in the cavity [21]. Furthermore, because of the comb modes being rigorously equally spaced [5,28,29], the resonant dispersion of each Fabry–Perot mode is pasted onto the whole comb structure, resulting in an enhancement of the sensor response [22].

This passive synchronization and tailoring of frequency combs is expected to be an invaluable part of future projects relating to atomic state preparation and probing. The ability to access specific transitions is an obvious advantage; however, the fact that these tailored combs can also be designed to reduce or eliminate energy at wavelengths corresponding unwanted resonances allows for a great reduction in noise in atomic experiments. This is accomplished without resorting to expensive stabilized continuous wave sources.

It also provides a much more efficient source for these types of state probing projects as laser energy is focused into specific frequency modes, resulting in less power being wasted in unnecessary frequency spaces. Further development of tailored combs and self-stabilization could result in much more cost effective tunable sources capable of a much broader range of applications than the original mode-locked sources.

Finally, the precision tuning of these tailored combs has been shown to be predictable and repeatable.

## Data Availability

The data presented in this study are available on request from the corresponding author.
